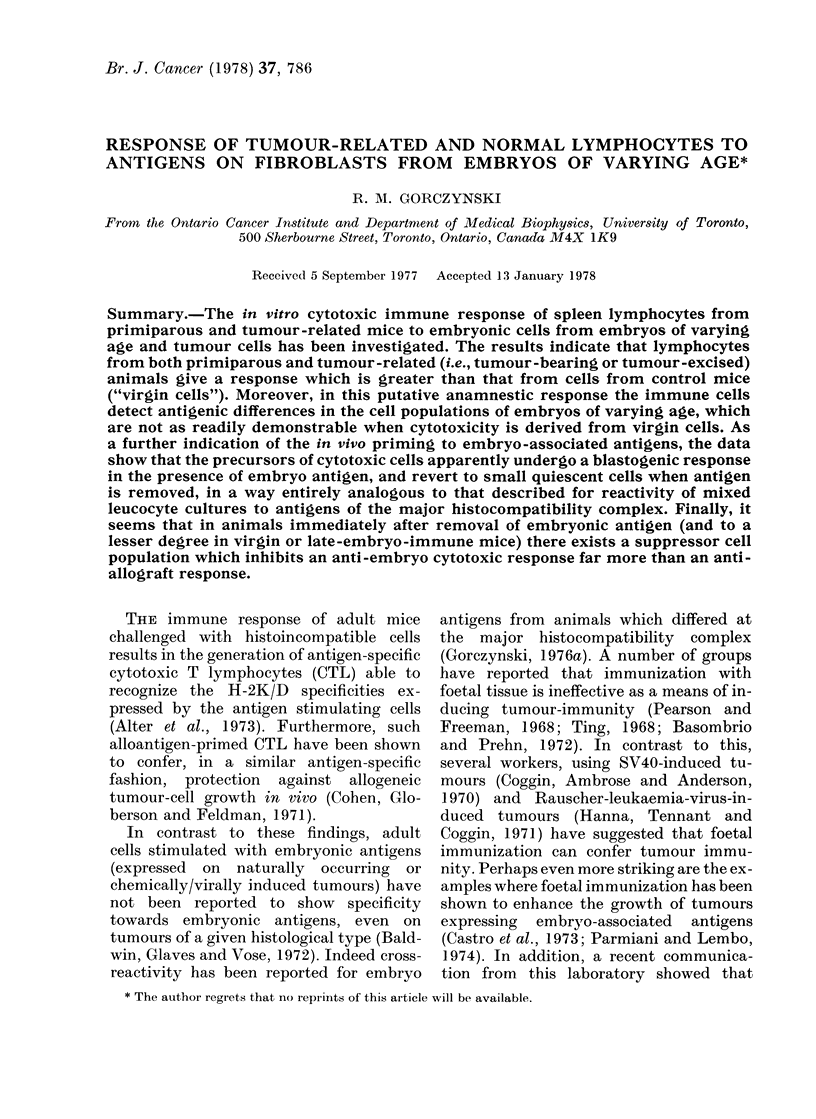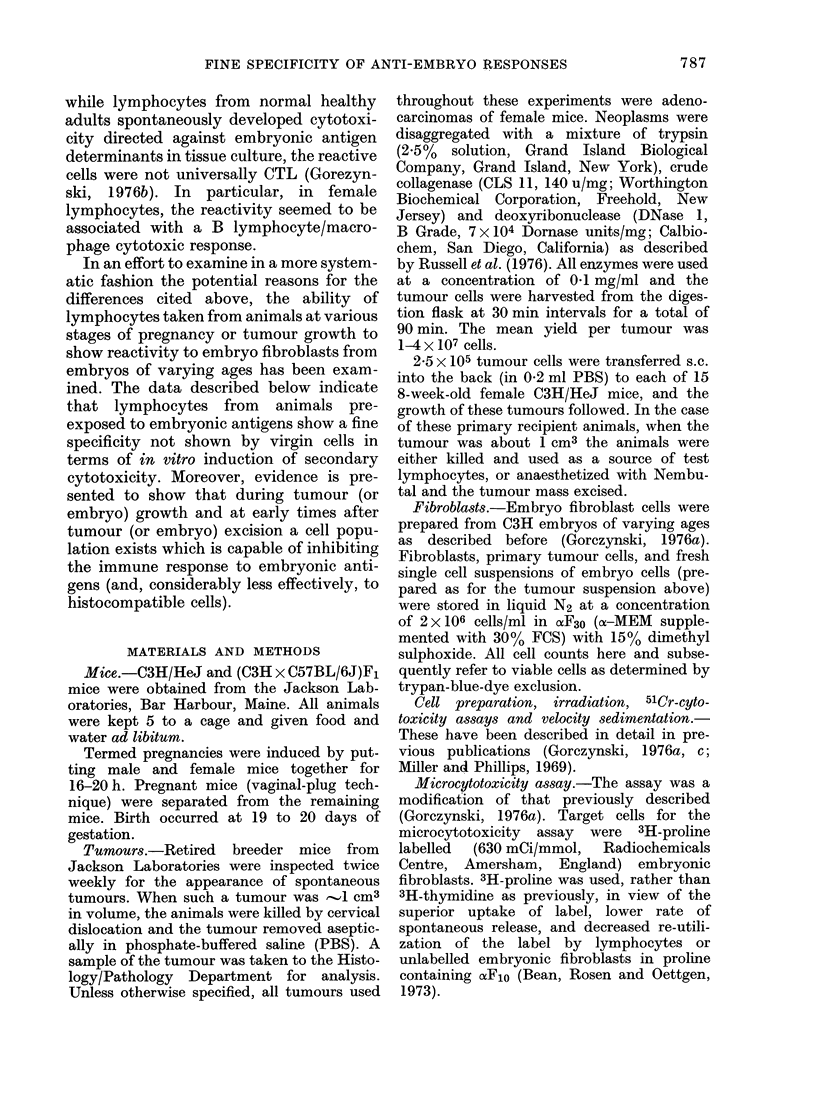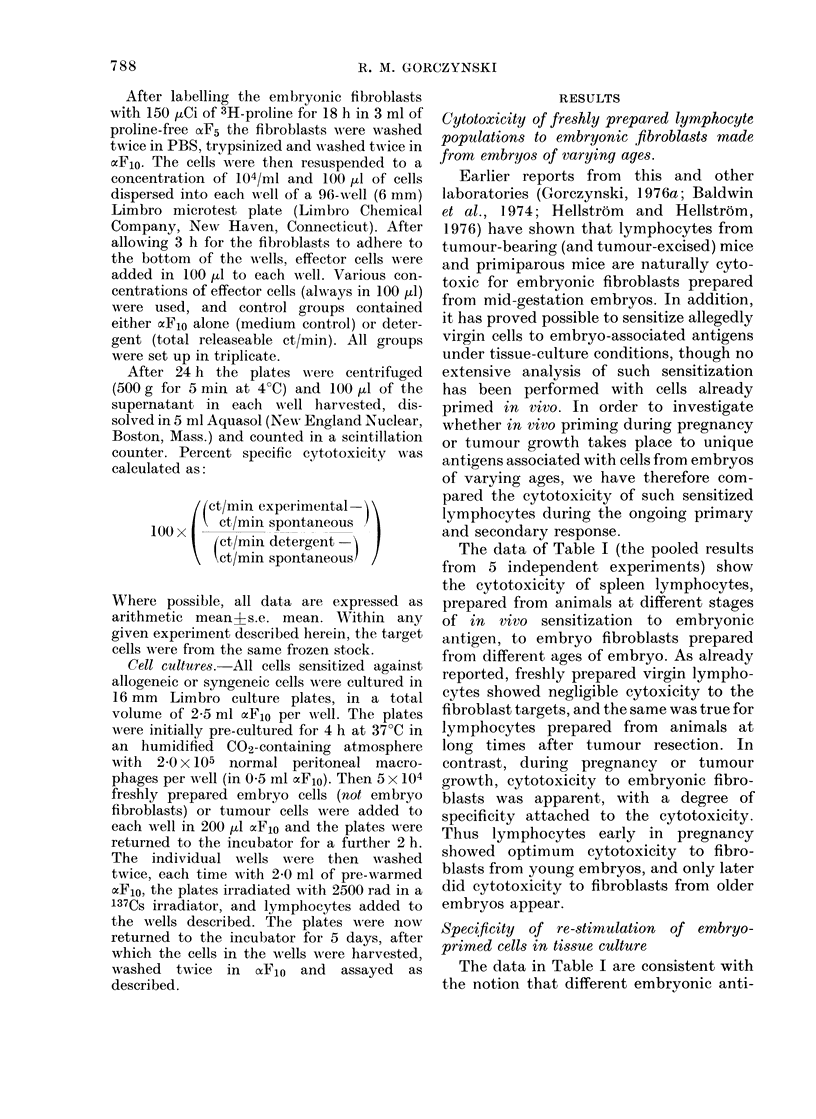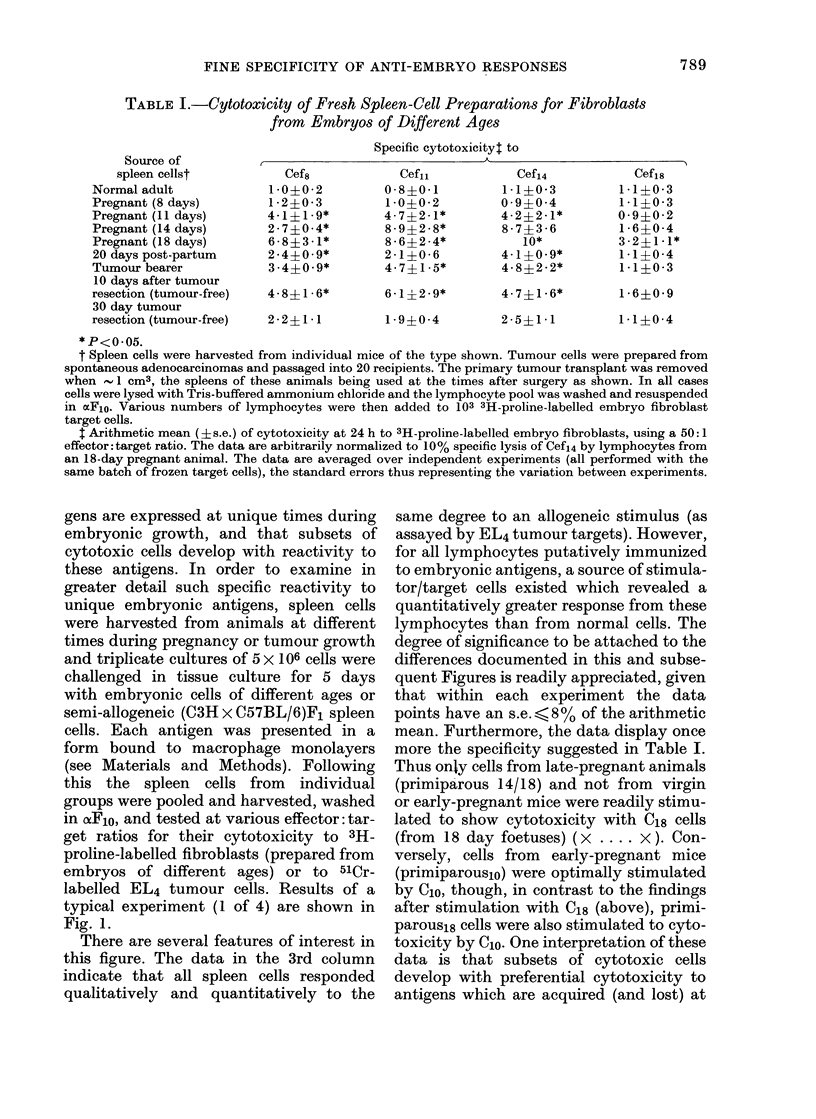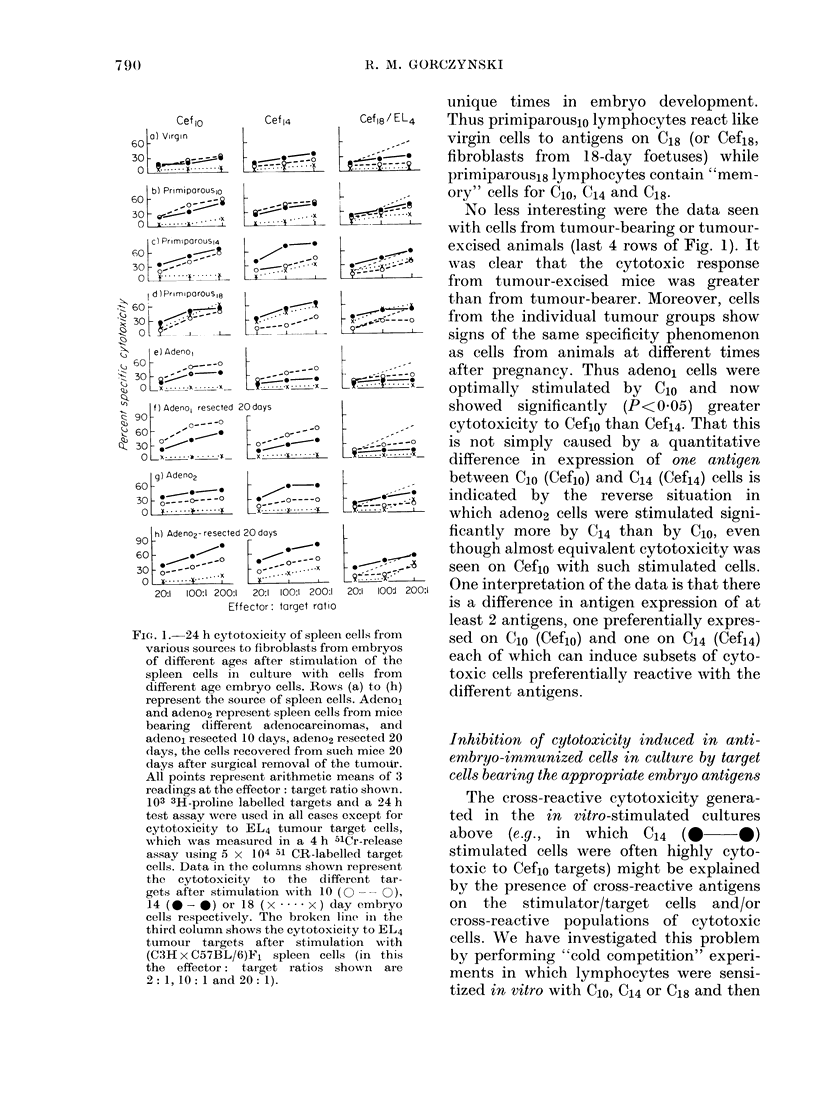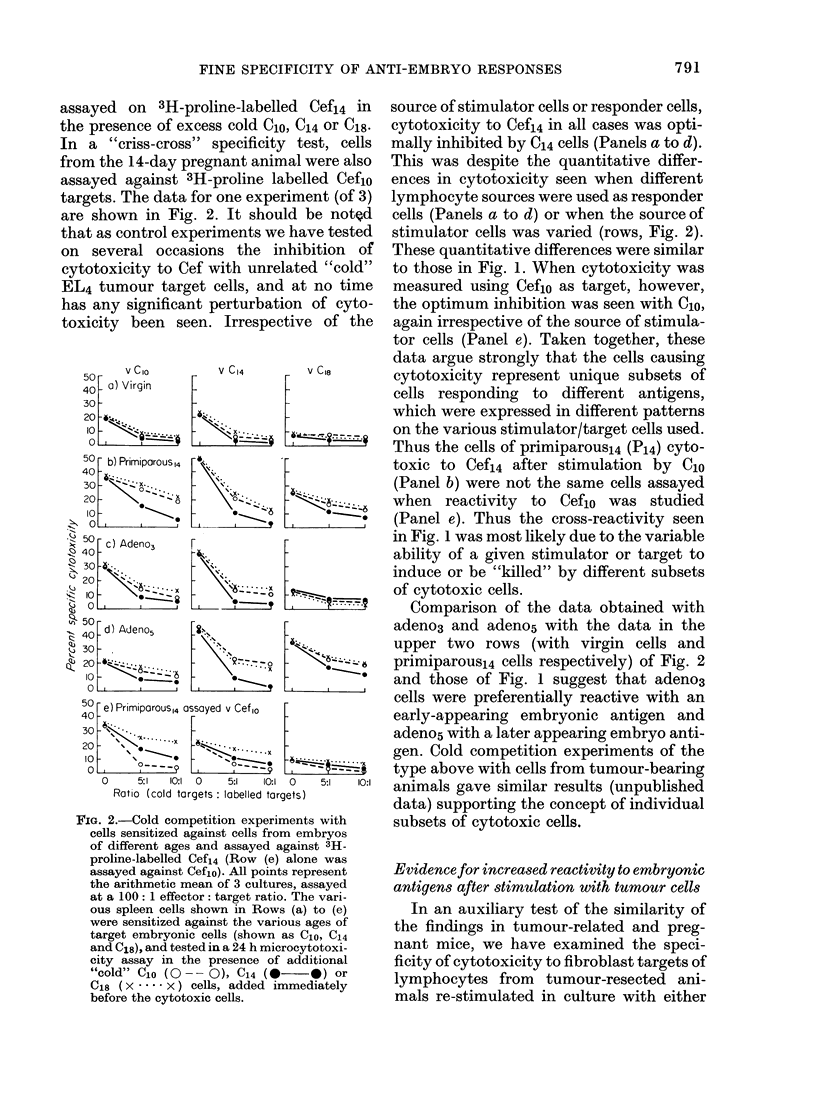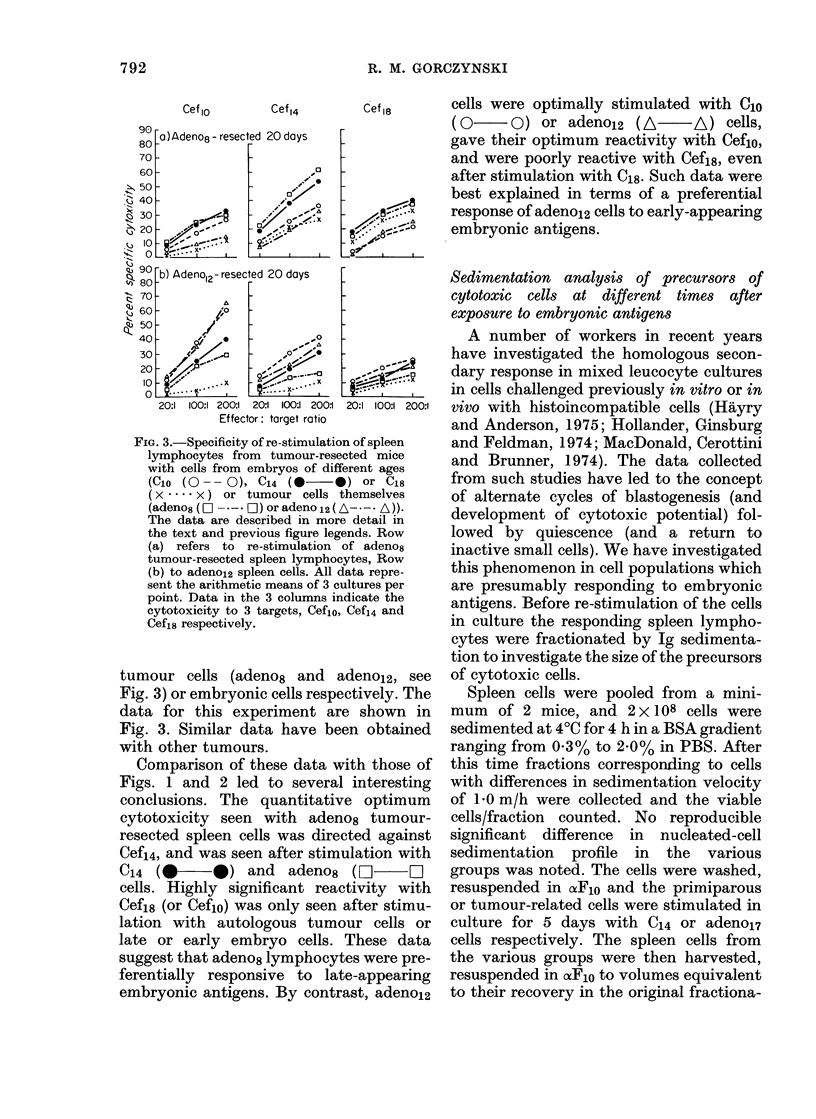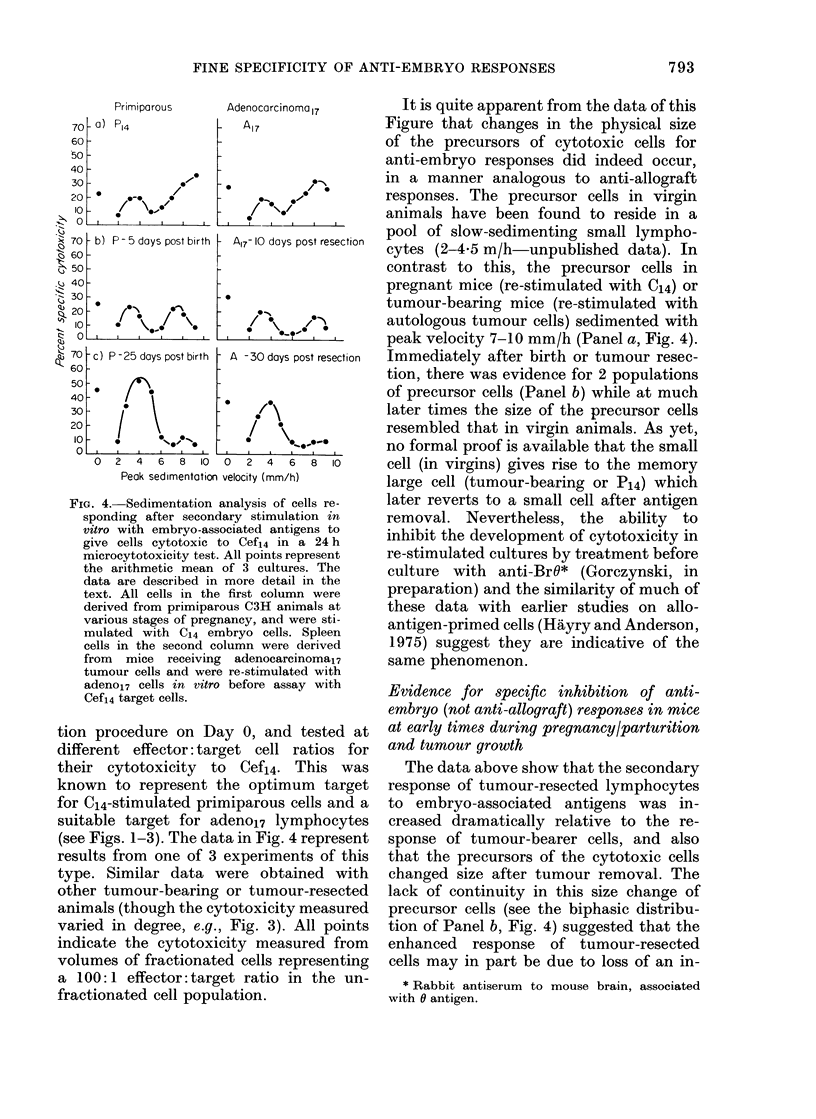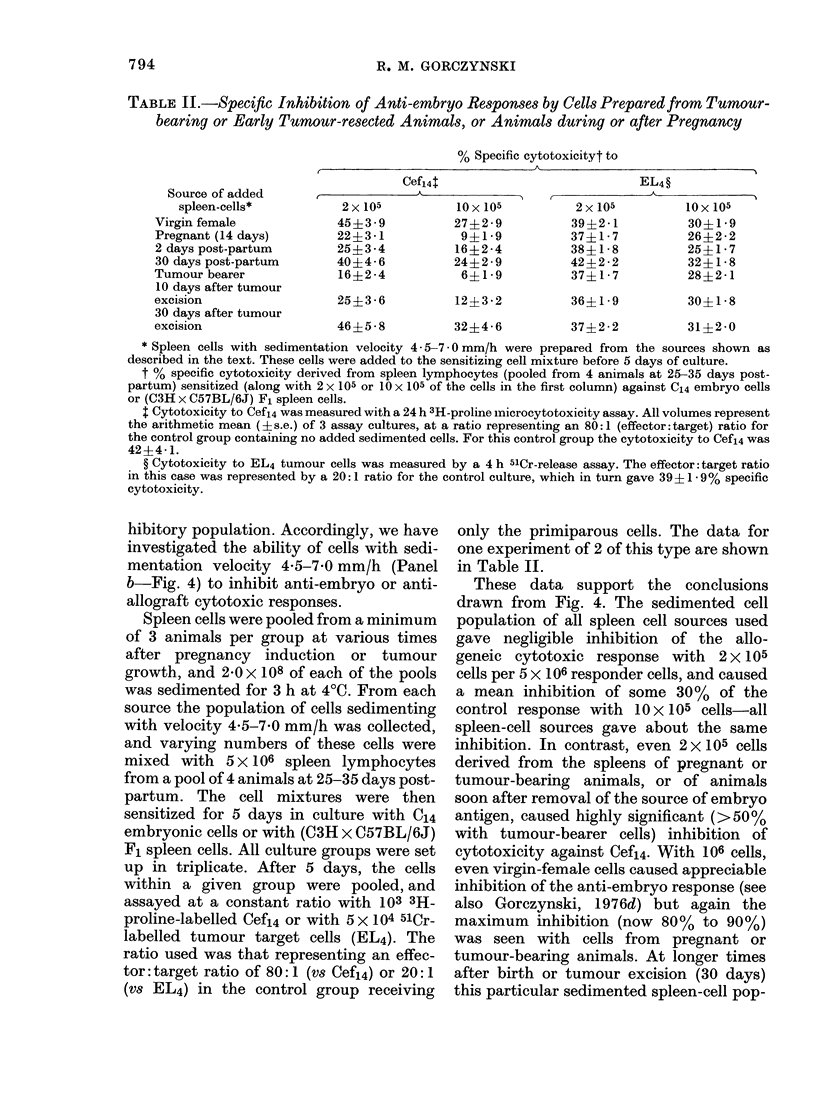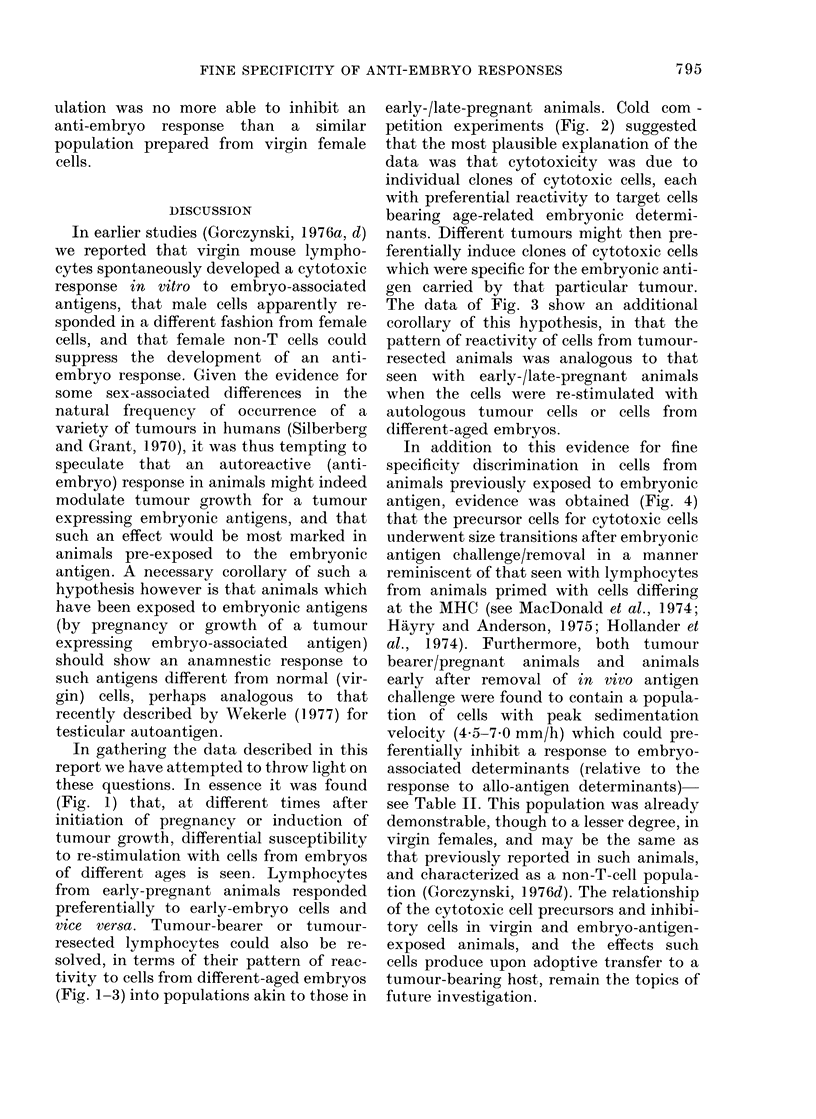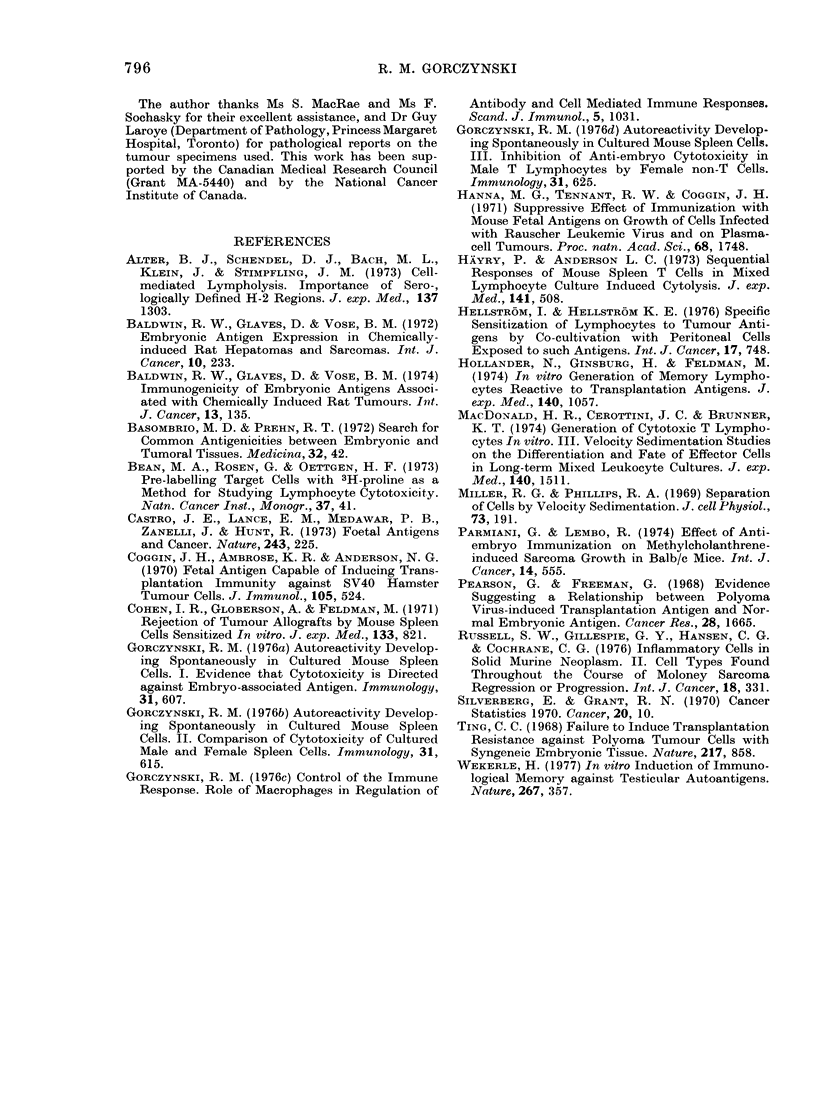# Response of tumour-related and normal lymphocytes to antigens on fibroblasts from embryos of varying age.

**DOI:** 10.1038/bjc.1978.117

**Published:** 1978-05

**Authors:** R. M. Gorczynski

## Abstract

The in vitro cytotoxic immune response of spleen lymphocytes from primiparous and tumour-related mice to embryonic cells from embryos of varying age and tumour cells has been investigated. The results indicate that lymphocytes from both primiparous and tumour-related (i.e., tumour-bearing or tumour-excised) animals give a response which is greater than that from cells from control mice ("virgin cells"). Moreover, in this putative anamnestic response the immune cells detect antigenic differences in the cell populations of embryos of varying age, which are not as readily demonstrable when cytotoxicity is derived from virgin cells. As a further indication of the in vivo priming to embryo-assoicated antigens, the data show that the precursors of cytotoxic cells apparently undergo a blastogenic response in the presence of embryo antigen, and revert to small quiescent cells when antigen is removed, in a way entirely analogous to that described for reactivity of mixed leucocyte cultures to antigens of the major histocompatibility complex. Finally, it seems that in animals immediately after removal of embryonic antigen (and to a lesser degree in virgin or late-embryo-immune mice) there exists a suppressor cell population which inhibits an anti-embryo cytotoxic response far more than an antiallograft response.


*Br. J. Cancer* (1978) **37**, 786

### RESPONSE OF TUMOUR-RELATED AND NORMAL LYMPHOCYTES TO ANTIGENS ON FIBROBLASTS FROM EMBRYOS OF VARYING AGE*

R. M. GORCZYNSKI

*From the Ontario Cancer Institute and Department of Medical Biophysics, University of Toronto,* 500 *Sherbourne Street, Toronto, Ontario, Canada M*4*X* 1*K*9

Received 5 September 1977 Accepted 13 January 1978

**Summary.—The *in vitro* cytotoxic immune response of spleen lymphocytes from primiparous and tumour-related mice to embryonic cells from embryos of varying age and tumour cells has been investigated. The results indicate that lymphocytes from both primiparous and tumour-related (*i.e.*, tumour-bearing or tumour-excised) animals give a response which is greater than that from cells from control mice ("virgin cells"). Moreover, in this putative anamnestic response the immune cells detect antigenic differences in the cell populations of embryos of varying age, which are not as readily demonstrable when cytotoxicity is derived from virgin cells. As a further indication of the *in vivo* priming to embryo-associated antigens, the data show that the precursors of cytotoxic cells apparently undergo a blastogenic response in the presence of embryo antigen, and revert to small quiescent cells when antigen is removed, in a way entirely analogous to that described for reactivity of mixed leucocyte cultures to antigens of the major histocompatibility complex. Finally, it seems that in animals immediately after removal of embryonic antigen (and to a lesser degree in virgin or late-embryo-immune mice) there exists a suppressor cell population which inhibits an anti-embryo cytotoxic response far more than an anti-allograft response.**

THE immune response of adult mice challenged with histoincompatible cells results in the generation of antigen-specific cytotoxic T lymphocytes (CTL) able to recognize the H-2K/D specificities expressed by the antigen stimulating cells (Alter *et al.*, 1973). Furthermore, such alloantigen-primed CTL have been shown to confer, in a similar antigen-specific fashion, protection against allogeneic tumour-cell growth *in vivo* (Cohen, Globerson and Feldman, 1971).

In contrast to these findings, adult cells stimulated with embryonic antigens (expressed on naturally occurring or chemically/virally induced tumours) have not been reported to show specificity towards embryonic antigens, even on tumours of a given histological type (Baldwin, Glaves and Vose, 1972). Indeed cross-reactivity has been reported for embryo antigens from animals which differed at the major histocompatibility complex (Gorczynski, 1976*a*). A number of groups have reported that immunization with foetal tissue is ineffective as a means of inducing tumour-immunity (Pearson and Freeman, 1968; Ting, 1968; Basombrio and Prehn, 1972). In contrast to this, several workers, using SV40-induced tumours (Coggin, Ambrose and Anderson, 1970) and Rauscher-leukaemia-virus-induced tumours (Hanna, Tennant and Coggin, 1971) have suggested that foetal immunization can confer tumour immunity. Perhaps even more striking are the examples where foetal immunization has been shown to enhance the growth of tumours expressing embryo-associated antigens (Castro *et al.*, 1973; Parmiani and Lembo, 1974). In addition, a recent communication from this laboratory showed that

* The author regrets that no reprints of this article will be available.

while lymphocytes from normal healthy adults spontaneously developed cytotoxicity directed against embryonic antigen determinants in tissue culture, the reactive cells were not universally CTL (Gorezynski, 1976*b*). In particular, in female lymphocytes, the reactivity seemed to be associated with a B lymphocyte/macrophage cytotoxic response.

In an effort to examine in a more systematic fashion the potential reasons for the differences cited above, the ability of lymphocytes taken from animals at various stages of pregnancy or tumour growth to show reactivity to embryo fibroblasts from embryos of varying ages has been examined. The data described below indicate that lymphocytes from animals pre-exposed to embryonic antigens show a fine specificity not shown by virgin cells in terms of *in vitro* induction of secondary cytotoxicity. Moreover, evidence is presented to show that during tumour (or embryo) growth and at early times after tumour (or embryo) excision a cell population exists which is capable of inhibiting the immune response to embryonic antigens (and, considerably less effectively, to histocompatible cells).

### MATERIALS AND METHODS

*Mice.*—C3H/HeJ and (C3H×C57BL/6J)$F_1$ mice were obtained from the Jackson Laboratories, Bar Harbour, Maine. All animals were kept 5 to a cage and given food and water *ad libitum.*

Termed pregnancies were induced by putting male and female mice together for 16–20 h. Pregnant mice (vaginal-plug technique) were separated from the remaining mice. Birth occurred at 19 to 20 days of gestation.

*Tumours.*—Retired breeder mice from Jackson Laboratories were inspected twice weekly for the appearance of spontaneous tumours. When such a tumour was $\sim$1 cm$^3$ in volume, the animals were killed by cervical dislocation and the tumour removed aseptically in phosphate-buffered saline (PBS). A sample of the tumour was taken to the Histology/Pathology Department for analysis. Unless otherwise specified, all tumours used throughout these experiments were adenocarcinomas of female mice. Neoplasms were disaggregated with a mixture of trypsin (2·5% solution, Grand Island Biological Company, Grand Island, New York), crude collagenase (CLS 11, 140 u/mg; Worthington Biochemical Corporation, Freehold, New Jersey) and deoxyribonuclease (DNase 1, B Grade, $7\times10^4$ Dornase units/mg; Calbiochem, San Diego, California) as described by Russell *et al.* (1976). All enzymes were used at a concentration of 0·1 mg/ml and the tumour cells were harvested from the digestion flask at 30 min intervals for a total of 90 min. The mean yield per tumour was $1–4\times10^7$ cells.

$2·5\times10^5$ tumour cells were transferred s.c. into the back (in 0·2 ml PBS) to each of 15 8-week-old female C3H/HeJ mice, and the growth of these tumours followed. In the case of these primary recipient animals, when the tumour was about 1 cm$^3$ the animals were either killed and used as a source of test lymphocytes, or anaesthetized with Nembutal and the tumour mass excised.

*Fibroblasts.*—Embryo fibroblast cells were prepared from C3H embryos of varying ages as described before (Gorczynski, 1976*a*). Fibroblasts, primary tumour cells, and fresh single cell suspensions of embryo cells (prepared as for the tumour suspension above) were stored in liquid $N_2$ at a concentration of $2\times10^6$ cells/ml in $\alpha F_{30}$ (α–MEM supplemented with 30% FCS) with 15% dimethyl sulphoxide. All cell counts here and subsequently refer to viable cells as determined by trypan-blue-dye exclusion.

*Cell preparation, irradiation, $^{51}$Cr-cytotoxicity assays and velocity sedimentation.*—These have been described in detail in previous publications (Gorczynski, 1976*a*, *c*; Miller and Phillips, 1969).

*Microcytotoxicity assay.*—The assay was a modification of that previously described (Gorczynski, 1976*a*). Target cells for the microcytotoxicity assay were $^3$H-proline labelled (630 mCi/mmol, Radiochemicals Centre, Amersham, England) embryonic fibroblasts. $^3$H-proline was used, rather than $^3$H-thymidine as previously, in view of the superior uptake of label, lower rate of spontaneous release, and decreased re-utilization of the label by lymphocytes or unlabelled embryonic fibroblasts in proline containing $\alpha F_{10}$ (Bean, Rosen and Oettgen, 1973).

After labelling the embryonic fibroblasts with 150 $\mu$Ci of $^3$H-proline for 18 h in 3 ml of proline-free $\alpha F_5$ the fibroblasts were washed twice in PBS, trypsinized and washed twice in $\alpha F_{10}$. The cells were then resuspended to a concentration of $10^4$/ml and 100 $\mu$l of cells dispersed into each well of a 96-well (6 mm) Limbro microtest plate (Limbro Chemical Company, New Haven, Connecticut). After allowing 3 h for the fibroblasts to adhere to the bottom of the wells, effector cells were added in 100 $\mu$l to each well. Various concentrations of effector cells (always in 100 $\mu$l) were used, and control groups contained either $\alpha F_{10}$ alone (medium control) or detergent (total releaseable ct/min). All groups were set up in triplicate.

After 24 h the plates were centrifuged (500 g for 5 min at 4°C) and 100 $\mu$l of the supernatant in each well harvested, dissolved in 5 ml Aquasol (New England Nuclear, Boston, Mass.) and counted in a scintillation counter. Percent specific cytotoxicity was calculated as:

$$100 \times \left( \frac{\begin{pmatrix}\text{ct/min experimental}- \\ \text{ct/min spontaneous}\end{pmatrix}}{\begin{pmatrix}\text{ct/min detergent}- \\ \text{ct/min spontaneous}\end{pmatrix}} \right)$$

Where possible, all data are expressed as arithmetic mean$\pm$s.e. mean. Within any given experiment described herein, the target cells were from the same frozen stock.

*Cell cultures.*—All cells sensitized against allogeneic or syngeneic cells were cultured in 16 mm Limbro culture plates, in a total volume of 2·5 ml $\alpha F_{10}$ per well. The plates were initially pre-cultured for 4 h at 37°C in an humidified $CO_2$-containing atmosphere with $2{\cdot}0 \times 10^5$ normal peritoneal macrophages per well (in 0·5 ml $\alpha F_{10}$). Then $5 \times 10^4$ freshly prepared embryo cells (*not* embryo fibroblasts) or tumour cells were added to each well in 200 $\mu$l $\alpha F_{10}$ and the plates were returned to the incubator for a further 2 h. The individual wells were then washed twice, each time with 2·0 ml of pre-warmed $\alpha F_{10}$, the plates irradiated with 2500 rad in a $^{137}$Cs irradiator, and lymphocytes added to the wells described. The plates were now returned to the incubator for 5 days, after which the cells in the wells were harvested, washed twice in $\alpha F_{10}$ and assayed as described.

### RESULTS

*Cytotoxicity of freshly prepared lymphocyte populations to embryonic fibroblasts made from embryos of varying ages.*

Earlier reports from this and other laboratories (Gorczynski, 1976*a*; Baldwin *et al.*, 1974; Hellström and Hellström, 1976) have shown that lymphocytes from tumour-bearing (and tumour-excised) mice and primiparous mice are naturally cytotoxic for embryonic fibroblasts prepared from mid-gestation embryos. In addition, it has proved possible to sensitize allegedly virgin cells to embryo-associated antigens under tissue-culture conditions, though no extensive analysis of such sensitization has been performed with cells already primed *in vivo*. In order to investigate whether *in vivo* priming during pregnancy or tumour growth takes place to unique antigens associated with cells from embryos of varying ages, we have therefore compared the cytotoxicity of such sensitized lymphocytes during the ongoing primary and secondary response.

The data of Table I (the pooled results from 5 independent experiments) show the cytotoxicity of spleen lymphocytes, prepared from animals at different stages of *in vivo* sensitization to embryonic antigen, to embryo fibroblasts prepared from different ages of embryo. As already reported, freshly prepared virgin lymphocytes showed negligible cytoxicity to the fibroblast targets, and the same was true for lymphocytes prepared from animals at long times after tumour resection. In contrast, during pregnancy or tumour growth, cytotoxicity to embryonic fibroblasts was apparent, with a degree of specificity attached to the cytotoxicity. Thus lymphocytes early in pregnancy showed optimum cytotoxicity to fibroblasts from young embryos, and only later did cytotoxicity to fibroblasts from older embryos appear.

*Specificity of re-stimulation of embryo-primed cells in tissue culture*

The data in Table I are consistent with the notion that different embryonic anti-

TABLE I.—*Cytotoxicity of Fresh Spleen-Cell Preparations for Fibroblasts from Embryos of Different Ages*

| Source of spleen cells† | Specific cytotoxicity‡ to | | | |
|---|---|---|---|---|
| | $Cef_8$ | $Cef_{11}$ | $Cef_{14}$ | $Cef_{18}$ |
| Normal adult | 1·0±0·2 | 0·8±0·1 | 1·1±0·3 | 1·1±0·3 |
| Pregnant (8 days) | 1·2±0·3 | 1·0±0·2 | 0·9±0·4 | 1·1±0·3 |
| Pregnant (11 days) | 4·1±1·9* | 4·7±2·1* | 4·2±2·1* | 0·9±0·2 |
| Pregnant (14 days) | 2·7±0·4* | 8·9±2·8* | 8·7±3·6 | 1·6±0·4 |
| Pregnant (18 days) | 6·8±3·1* | 8·6±2·4* | 10* | 3·2±1·1* |
| 20 days post-partum | 2·4±0·9* | 2·1±0·6 | 4·1±0·9* | 1·1±0·4 |
| Tumour bearer | 3·4±0·9* | 4·7±1·5* | 4·8±2·2* | 1·1±0·3 |
| 10 days after tumour resection (tumour-free) | 4·8±1·6* | 6·1±2·9* | 4·7±1·6* | 1·6±0·9 |
| 30 day tumour resection (tumour-free) | 2·2±1·1 | 1·9±0·4 | 2·5±1·1 | 1·1±0·4 |

* $P<0·05$.

† Spleen cells were harvested from individual mice of the type shown. Tumour cells were prepared from spontaneous adenocarcinomas and passaged into 20 recipients. The primary tumour transplant was removed when $\sim 1$ cm$^3$, the spleens of these animals being used at the times after surgery as shown. In all cases cells were lysed with Tris-buffered ammonium chloride and the lymphocyte pool was washed and resuspended in $\alpha F_{10}$. Various numbers of lymphocytes were then added to $10^3$ $^3$H-proline-labelled embryo fibroblast target cells.

‡ Arithmetic mean (±s.e.) of cytotoxicity at 24 h to $^3$H-proline-labelled embryo fibroblasts, using a 50:1 effector:target ratio. The data are arbitrarily normalized to 10% specific lysis of $Cef_{14}$ by lymphocytes from an 18-day pregnant animal. The data are averaged over independent experiments (all performed with the same batch of frozen target cells), the standard errors thus representing the variation between experiments.

gens are expressed at unique times during embryonic growth, and that subsets of cytotoxic cells develop with reactivity to these antigens. In order to examine in greater detail such specific reactivity to unique embryonic antigens, spleen cells were harvested from animals at different times during pregnancy or tumour growth and triplicate cultures of $5\times10^6$ cells were challenged in tissue culture for 5 days with embryonic cells of different ages or semi-allogeneic (C3H×C57BL/6)$F_1$ spleen cells. Each antigen was presented in a form bound to macrophage monolayers (see Materials and Methods). Following this the spleen cells from individual groups were pooled and harvested, washed in $\alpha F_{10}$, and tested at various effector:target ratios for their cytotoxicity to $^3$H-proline-labelled fibroblasts (prepared from embryos of different ages) or to $^{51}$Cr-labelled $EL_4$ tumour cells. Results of a typical experiment (1 of 4) are shown in Fig. 1.

There are several features of interest in this figure. The data in the 3rd column indicate that all spleen cells responded qualitatively and quantitatively to the same degree to an allogeneic stimulus (as assayed by $EL_4$ tumour targets). However, for all lymphocytes putatively immunized to embryonic antigens, a source of stimulator/target cells existed which revealed a quantitatively greater response from these lymphocytes than from normal cells. The degree of significance to be attached to the differences documented in this and subsequent Figures is readily appreciated, given that within each experiment the data points have an s.e.$\leqslant 8\%$ of the arithmetic mean. Furthermore, the data display once more the specificity suggested in Table I. Thus only cells from late-pregnant animals (primiparous 14/18) and not from virgin or early-pregnant mice were readily stimulated to show cytotoxicity with $C_{18}$ cells (from 18 day foetuses) (× . . . . ×). Conversely, cells from early-pregnant mice (primiparous$_{10}$) were optimally stimulated by $C_{10}$, though, in contrast to the findings after stimulation with $C_{18}$ (above), primiparous$_{18}$ cells were also stimulated to cytotoxicity by $C_{10}$. One interpretation of these data is that subsets of cytotoxic cells develop with preferential cytotoxicity to antigens which are acquired (and lost) at

FIG. 1.—24 h cytotoxicity of spleen cells from various sources to fibroblasts from embryos of different ages after stimulation of the spleen cells in culture with cells from different age embryo cells. Rows (a) to (h) represent the source of spleen cells. $Adeno_1$ and $adeno_2$ represent spleen cells from mice bearing different adenocarcinomas, and $adeno_1$ resected 10 days, $adeno_2$ resected 20 days, the cells recovered from such mice 20 days after surgical removal of the tumour. All points represent arithmetic means of 3 readings at the effector : target ratio shown. $10^3$ $^3H$-proline labelled targets and a 24 h test assay were used in all cases except for cytotoxicity to $EL_4$ tumour target cells, which was measured in a 4 h $^{51}Cr$-release assay using $5 \times 10^4$ $^{51}$ CR-labelled target cells. Data in the columns shown represent the cytotoxicity to the different targets after stimulation with 10 (○ – – ○), 14 (● – ●) or 18 (× · · · · ×) day embryo cells respectively. The broken line in the third column shows the cytotoxicity to $EL_4$ tumour targets after stimulation with $(C3H \times C57BL/6)F_1$ spleen cells (in this the effector : target ratios shown are 2 : 1, 10 : 1 and 20 : 1).

unique times in embryo development. Thus $primiparous_{10}$ lymphocytes react like virgin cells to antigens on $C_{18}$ (or $Cef_{18}$, fibroblasts from 18-day foetuses) while $primiparous_{18}$ lymphocytes contain "memory" cells for $C_{10}$, $C_{14}$ and $C_{18}$.

No less interesting were the data seen with cells from tumour-bearing or tumour-excised animals (last 4 rows of Fig. 1). It was clear that the cytotoxic response from tumour-excised mice was greater than from tumour-bearer. Moreover, cells from the individual tumour groups show signs of the same specificity phenomenon as cells from animals at different times after pregnancy. Thus $adeno_1$ cells were optimally stimulated by $C_{10}$ and now showed significantly ($P<0{\cdot}05$) greater cytotoxicity to $Cef_{10}$ than $Cef_{14}$. That this is not simply caused by a quantitative difference in expression of *one antigen* between $C_{10}$ ($Cef_{10}$) and $C_{14}$ ($Cef_{14}$) cells is indicated by the reverse situation in which $adeno_2$ cells were stimulated significantly more by $C_{14}$ than by $C_{10}$, even though almost equivalent cytotoxicity was seen on $Cef_{10}$ with such stimulated cells. One interpretation of the data is that there is a difference in antigen expression of at least 2 antigens, one preferentially expressed on $C_{10}$ ($Cef_{10}$) and one on $C_{14}$ ($Cef_{14}$) each of which can induce subsets of cytotoxic cells preferentially reactive with the different antigens.

### *Inhibition of cytotoxicity induced in anti-embryo-immunized cells in culture by target cells bearing the appropriate embryo antigens*

The cross-reactive cytotoxicity generated in the *in vitro*-stimulated cultures above (*e.g.*, in which $C_{14}$ (●——●) stimulated cells were often highly cytotoxic to $Cef_{10}$ targets) might be explained by the presence of cross-reactive antigens on the stimulator/target cells and/or cross-reactive populations of cytotoxic cells. We have investigated this problem by performing "cold competition" experiments in which lymphocytes were sensitized *in vitro* with $C_{10}$, $C_{14}$ or $C_{18}$ and then assayed on $^3$H-proline-labelled $Cef_{14}$ in the presence of excess cold $C_{10}$, $C_{14}$ or $C_{18}$. In a "criss-cross" specificity test, cells from the 14-day pregnant animal were also assayed against $^3$H-proline labelled $Cef_{10}$ targets. The data for one experiment (of 3) are shown in Fig. 2. It should be noted that as control experiments we have tested on several occasions the inhibition of cytotoxicity to Cef with unrelated "cold" $EL_4$ tumour target cells, and at no time has any significant perturbation of cytotoxicity been seen. Irrespective of the source of stimulator cells or responder cells, cytotoxicity to $Cef_{14}$ in all cases was optimally inhibited by $C_{14}$ cells (Panels *a* to *d*). This was despite the quantitative differences in cytotoxicity seen when different lymphocyte sources were used as responder cells (Panels *a* to *d*) or when the source of stimulator cells was varied (rows, Fig. 2). These quantitative differences were similar to those in Fig. 1. When cytotoxicity was measured using $Cef_{10}$ as target, however, the optimum inhibition was seen with $C_{10}$, again irrespective of the source of stimulator cells (Panel *e*). Taken together, these data argue strongly that the cells causing cytotoxicity represent unique subsets of cells responding to different antigens, which were expressed in different patterns on the various stimulator/target cells used. Thus the cells of primiparous$_{14}$ ($P_{14}$) cytotoxic to $Cef_{14}$ after stimulation by $C_{10}$ (Panel *b*) were not the same cells assayed when reactivity to $Cef_{10}$ was studied (Panel *e*). Thus the cross-reactivity seen in Fig. 1 was most likely due to the variable ability of a given stimulator or target to induce or be "killed" by different subsets of cytotoxic cells.

FIG. 2.—Cold competition experiments with cells sensitized against cells from embryos of different ages and assayed against $^3$H-proline-labelled $Cef_{14}$ (Row (e) alone was assayed against $Cef_{10}$). All points represent the arithmetic mean of 3 cultures, assayed at a 100 : 1 effector : target ratio. The various spleen cells shown in Rows (a) to (e) were sensitized against the various ages of target embryonic cells (shown as $C_{10}$, $C_{14}$ and $C_{18}$), and tested in a 24 h microcytotoxicity assay in the presence of additional "cold" $C_{10}$ (○ – – ○), $C_{14}$ (●——●) or $C_{18}$ (× · · · · ×) cells, added immediately before the cytotoxic cells.

Comparison of the data obtained with adeno$_3$ and adeno$_5$ with the data in the upper two rows (with virgin cells and primiparous$_{14}$ cells respectively) of Fig. 2 and those of Fig. 1 suggest that adeno$_3$ cells were preferentially reactive with an early-appearing embryonic antigen and adeno$_5$ with a later appearing embryo antigen. Cold competition experiments of the type above with cells from tumour-bearing animals gave similar results (unpublished data) supporting the concept of individual subsets of cytotoxic cells.

### *Evidence for increased reactivity to embryonic antigens after stimulation with tumour cells*

In an auxiliary test of the similarity of the findings in tumour-related and pregnant mice, we have examined the specificity of cytotoxicity to fibroblast targets of lymphocytes from tumour-resected animals re-stimulated in culture with either

FIG. 3.—Specificity of re-stimulation of spleen lymphocytes from tumour-resected mice with cells from embryos of different ages ($C_{10}$ (○ - - ○), $C_{14}$ (●——●) or $C_{18}$ (× · · · ×) or tumour cells themselves (adeno$_8$ (□ —·— □) or adeno$_{12}$ (△—·—△)). The data are described in more detail in the text and previous figure legends. Row (a) refers to re-stimulation of adeno$_8$ tumour-resected spleen lymphocytes, Row (b) to adeno$_{12}$ spleen cells. All data represent the arithmetic means of 3 cultures per point. Data in the 3 columns indicate the cytotoxicity to 3 targets, Cef$_{10}$, Cef$_{14}$ and Cef$_{18}$ respectively.

tumour cells (adeno$_8$ and adeno$_{12}$, see Fig. 3) or embryonic cells respectively. The data for this experiment are shown in Fig. 3. Similar data have been obtained with other tumours.

Comparison of these data with those of Figs. 1 and 2 led to several interesting conclusions. The quantitative optimum cytotoxicity seen with adeno$_8$ tumour-resected spleen cells was directed against Cef$_{14}$, and was seen after stimulation with $C_{14}$ (●——●) and adeno$_8$ (□——□ cells. Highly significant reactivity with Cef$_{18}$ (or Cef$_{10}$) was only seen after stimulation with autologous tumour cells or late or early embryo cells. These data suggest that adeno$_8$ lymphocytes were preferentially responsive to late-appearing embryonic antigens. By contrast, adeno$_{12}$ cells were optimally stimulated with $C_{10}$ (○——○) or adeno$_{12}$ (△——△) cells, gave their optimum reactivity with Cef$_{10}$, and were poorly reactive with Cef$_{18}$, even after stimulation with $C_{18}$. Such data were best explained in terms of a preferential response of adeno$_{12}$ cells to early-appearing embryonic antigens.

*Sedimentation analysis of precursors of cytotoxic cells at different times after exposure to embryonic antigens*

A number of workers in recent years have investigated the homologous secondary response in mixed leucocyte cultures in cells challenged previously *in vitro* or *in vivo* with histoincompatible cells (Häyry and Anderson, 1975; Hollander, Ginsburg and Feldman, 1974; MacDonald, Cerottini and Brunner, 1974). The data collected from such studies have led to the concept of alternate cycles of blastogenesis (and development of cytotoxic potential) followed by quiescence (and a return to inactive small cells). We have investigated this phenomenon in cell populations which are presumably responding to embryonic antigens. Before re-stimulation of the cells in culture the responding spleen lymphocytes were fractionated by Ig sedimentation to investigate the size of the precursors of cytotoxic cells.

Spleen cells were pooled from a minimum of 2 mice, and $2 \times 10^8$ cells were sedimented at 4°C for 4 h in a BSA gradient ranging from 0·3% to 2·0% in PBS. After this time fractions corresponding to cells with differences in sedimentation velocity of 1·0 m/h were collected and the viable cells/fraction counted. No reproducible significant difference in nucleated-cell sedimentation profile in the various groups was noted. The cells were washed, resuspended in $\alpha F_{10}$ and the primiparous or tumour-related cells were stimulated in culture for 5 days with $C_{14}$ or adeno$_{17}$ cells respectively. The spleen cells from the various groups were then harvested, resuspended in $\alpha F_{10}$ to volumes equivalent to their recovery in the original fractiona-

FIG. 4.—Sedimentation analysis of cells responding after secondary stimulation *in vitro* with embryo-associated antigens to give cells cytotoxic to $Cef_{14}$ in a 24 h microcytotoxicity test. All points represent the arithmetic mean of 3 cultures. The data are described in more detail in the text. All cells in the first column were derived from primiparous C3H animals at various stages of pregnancy, and were stimulated with $C_{14}$ embryo cells. Spleen cells in the second column were derived from mice receiving $adenocarcinoma_{17}$ tumour cells and were re-stimulated with $adeno_{17}$ cells *in vitro* before assay with $Cef_{14}$ target cells.

tion procedure on Day 0, and tested at different effector: target cell ratios for their cytotoxicity to $Cef_{14}$. This was known to represent the optimum target for $C_{14}$-stimulated primiparous cells and a suitable target for $adeno_{17}$ lymphocytes (see Figs. 1–3). The data in Fig. 4 represent results from one of 3 experiments of this type. Similar data were obtained with other tumour-bearing or tumour-resected animals (though the cytotoxicity measured varied in degree, *e.g.*, Fig. 3). All points indicate the cytotoxicity measured from volumes of fractionated cells representing a 100:1 effector: target ratio in the unfractionated cell population.

It is quite apparent from the data of this Figure that changes in the physical size of the precursors of cytotoxic cells for anti-embryo responses did indeed occur, in a manner analogous to anti-allograft responses. The precursor cells in virgin animals have been found to reside in a pool of slow-sedimenting small lymphocytes (2–4·5 m/h—unpublished data). In contrast to this, the precursor cells in pregnant mice (re-stimulated with $C_{14}$) or tumour-bearing mice (re-stimulated with autologous tumour cells) sedimented with peak velocity 7–10 mm/h (Panel *a*, Fig. 4). Immediately after birth or tumour resection, there was evidence for 2 populations of precursor cells (Panel *b*) while at much later times the size of the precursor cells resembled that in virgin animals. As yet, no formal proof is available that the small cell (in virgins) gives rise to the memory large cell (tumour-bearing or $P_{14}$) which later reverts to a small cell after antigen removal. Nevertheless, the ability to inhibit the development of cytotoxicity in re-stimulated cultures by treatment before culture with anti-Br$\theta$* (Gorczynski, in preparation) and the similarity of much of these data with earlier studies on allo-antigen-primed cells (Häyry and Anderson, 1975) suggest they are indicative of the same phenomenon.

*Evidence for specific inhibition of anti-embryo (not anti-allograft) responses in mice at early times during pregnancy/parturition and tumour growth*

The data above show that the secondary response of tumour-resected lymphocytes to embryo-associated antigens was increased dramatically relative to the response of tumour-bearer cells, and also that the precursors of the cytotoxic cells changed size after tumour removal. The lack of continuity in this size change of precursor cells (see the biphasic distribution of Panel *b*, Fig. 4) suggested that the enhanced response of tumour-resected cells may in part be due to loss of an in-

* Rabbit antiserum to mouse brain, associated with $\theta$ antigen.

TABLE II.—*Specific Inhibition of Anti-embryo Responses by Cells Prepared from Tumour-bearing or Early Tumour-resected Animals, or Animals during or after Pregnancy*

| | % Specific cytotoxicity† to | | | |
|---|---|---|---|---|
| | $Cef_{14}$‡ | | $EL_4$§ | |
| Source of added spleen-cells* | $2\times10^5$ | $10\times10^5$ | $2\times10^5$ | $10\times10^5$ |
| Virgin female | 45±3·9 | 27±2·9 | 39±2·1 | 30±1·9 |
| Pregnant (14 days) | 22±3·1 | 9±1·9 | 37±1·7 | 26±2·2 |
| 2 days post-partum | 25±3·4 | 16±2·4 | 38±1·8 | 25±1·7 |
| 30 days post-partum | 40±4·6 | 24±2·9 | 42±2·2 | 32±1·8 |
| Tumour bearer | 16±2·4 | 6±1·9 | 37±1·7 | 28±2·1 |
| 10 days after tumour excision | 25±3·6 | 12±3·2 | 36±1·9 | 30±1·8 |
| 30 days after tumour excision | 46±5·8 | 32±4·6 | 37±2·2 | 31±2·0 |

* Spleen cells with sedimentation velocity 4·5–7·0 mm/h were prepared from the sources shown as described in the text. These cells were added to the sensitizing cell mixture before 5 days of culture.

† % specific cytotoxicity derived from spleen lymphocytes (pooled from 4 animals at 25–35 days post-partum) sensitized (along with $2\times10^5$ or $10\times10^5$ of the cells in the first column) against $C_{14}$ embryo cells or (C3H × C57BL/6J) $F_1$ spleen cells.

‡ Cytotoxicity to $Cef_{14}$ was measured with a 24 h $^3$H-proline microcytotoxicity assay. All volumes represent the arithmetic mean (±s.e.) of 3 assay cultures, at a ratio representing an 80:1 (effector:target) ratio for the control group containing no added sedimented cells. For this control group the cytotoxicity to $Cef_{14}$ was 42±4·1.

§ Cytotoxicity to $EL_4$ tumour cells was measured by a 4 h $^{51}$Cr-release assay. The effector:target ratio in this case was represented by a 20:1 ratio for the control culture, which in turn gave 39±1·9% specific cytotoxicity.

hibitory population. Accordingly, we have investigated the ability of cells with sedimentation velocity 4·5–7·0 mm/h (Panel *b*—Fig. 4) to inhibit anti-embryo or anti-allograft cytotoxic responses.

Spleen cells were pooled from a minimum of 3 animals per group at various times after pregnancy induction or tumour growth, and $2{\cdot}0\times10^8$ of each of the pools was sedimented for 3 h at 4°C. From each source the population of cells sedimenting with velocity 4·5–7·0 mm/h was collected, and varying numbers of these cells were mixed with $5\times10^6$ spleen lymphocytes from a pool of 4 animals at 25–35 days post-partum. The cell mixtures were then sensitized for 5 days in culture with $C_{14}$ embryonic cells or with (C3H × C57BL/6J) $F_1$ spleen cells. All culture groups were set up in triplicate. After 5 days, the cells within a given group were pooled, and assayed at a constant ratio with $10^3$ $^3$H-proline-labelled $Cef_{14}$ or with $5\times10^4$ $^{51}$Cr-labelled tumour target cells ($EL_4$). The ratio used was that representing an effector:target ratio of 80:1 (*vs* $Cef_{14}$) or 20:1 (*vs* $EL_4$) in the control group receiving only the primiparous cells. The data for one experiment of 2 of this type are shown in Table II.

These data support the conclusions drawn from Fig. 4. The sedimented cell population of all spleen cell sources used gave negligible inhibition of the allogeneic cytotoxic response with $2\times10^5$ cells per $5\times10^6$ responder cells, and caused a mean inhibition of some 30% of the control response with $10\times10^5$ cells—all spleen-cell sources gave about the same inhibition. In contrast, even $2\times10^5$ cells derived from the spleens of pregnant or tumour-bearing animals, or of animals soon after removal of the source of embryo antigen, caused highly significant (>50% with tumour-bearer cells) inhibition of cytotoxicity against $Cef_{14}$. With $10^6$ cells, even virgin-female cells caused appreciable inhibition of the anti-embryo response (see also Gorczynski, 1976*d*) but again the maximum inhibition (now 80% to 90%) was seen with cells from pregnant or tumour-bearing animals. At longer times after birth or tumour excision (30 days) this particular sedimented spleen-cell population was no more able to inhibit an anti-embryo response than a similar population prepared from virgin female cells.

### DISCUSSION

In earlier studies (Gorczynski, 1976*a*, *d*) we reported that virgin mouse lymphocytes spontaneously developed a cytotoxic response *in vitro* to embryo-associated antigens, that male cells apparently responded in a different fashion from female cells, and that female non-T cells could suppress the development of an anti-embryo response. Given the evidence for some sex-associated differences in the natural frequency of occurrence of a variety of tumours in humans (Silberberg and Grant, 1970), it was thus tempting to speculate that an autoreactive (anti-embryo) response in animals might indeed modulate tumour growth for a tumour expressing embryonic antigens, and that such an effect would be most marked in animals pre-exposed to the embryonic antigen. A necessary corollary of such a hypothesis however is that animals which have been exposed to embryonic antigens (by pregnancy or growth of a tumour expressing embryo-associated antigen) should show an anamnestic response to such antigens different from normal (virgin) cells, perhaps analogous to that recently described by Wekerle (1977) for testicular autoantigen.

In gathering the data described in this report we have attempted to throw light on these questions. In essence it was found (Fig. 1) that, at different times after initiation of pregnancy or induction of tumour growth, differential susceptibility to re-stimulation with cells from embryos of different ages is seen. Lymphocytes from early-pregnant animals responded preferentially to early-embryo cells and *vice versa*. Tumour-bearer or tumour-resected lymphocytes could also be resolved, in terms of their pattern of reactivity to cells from different-aged embryos (Fig. 1–3) into populations akin to those in early-/late-pregnant animals. Cold competition experiments (Fig. 2) suggested that the most plausible explanation of the data was that cytotoxicity was due to individual clones of cytotoxic cells, each with preferential reactivity to target cells bearing age-related embryonic determinants. Different tumours might then preferentially induce clones of cytotoxic cells which were specific for the embryonic antigen carried by that particular tumour. The data of Fig. 3 show an additional corollary of this hypothesis, in that the pattern of reactivity of cells from tumour-resected animals was analogous to that seen with early-/late-pregnant animals when the cells were re-stimulated with autologous tumour cells or cells from different-aged embryos.

In addition to this evidence for fine specificity discrimination in cells from animals previously exposed to embryonic antigen, evidence was obtained (Fig. 4) that the precursor cells for cytotoxic cells underwent size transitions after embryonic antigen challenge/removal in a manner reminiscent of that seen with lymphocytes from animals primed with cells differing at the MHC (see MacDonald *et al.*, 1974; Häyry and Anderson, 1975; Hollander *et al.*, 1974). Furthermore, both tumour bearer/pregnant animals and animals early after removal of *in vivo* antigen challenge were found to contain a population of cells with peak sedimentation velocity (4·5–7·0 mm/h) which could preferentially inhibit a response to embryo-associated determinants (relative to the response to allo-antigen determinants)—see Table II. This population was already demonstrable, though to a lesser degree, in virgin females, and may be the same as that previously reported in such animals, and characterized as a non-T-cell population (Gorczynski, 1976*d*). The relationship of the cytotoxic cell precursors and inhibitory cells in virgin and embryo-antigen-exposed animals, and the effects such cells produce upon adoptive transfer to a tumour-bearing host, remain the topics of future investigation.

The author thanks Ms S. MacRae and Ms F. Sochasky for their excellent assistance, and Dr Guy Laroye (Department of Pathology, Princess Margaret Hospital, Toronto) for pathological reports on the tumour specimens used. This work has been supported by the Canadian Medical Research Council (Grant MA-5440) and by the National Cancer Institute of Canada.

### REFERENCES

ALTER, B. J., SCHENDEL, D. J., BACH, M. L., KLEIN, J. & STIMPFLING, J. M. (1973) Cell-mediated Lympholysis. Importance of Serologically Defined H-2 Regions. *J. exp. Med.*, **137** 1303.

BALDWIN, R. W., GLAVES, D. & VOSE, B. M. (1972) Embryonic Antigen Expression in Chemically-induced Rat Hepatomas and Sarcomas. *Int. J. Cancer*, **10**, 233.

BALDWIN, R. W., GLAVES, D. & VOSE, B. M. (1974) Immunogenicity of Embryonic Antigens Associated with Chemically Induced Rat Tumours. *Int. J. Cancer*, **13**, 135.

BASOMBRIO, M. D. & PREHN, R. T. (1972) Search for Common Antigenicities between Embryonic and Tumoral Tissues. *Medicina*, **32**, 42.

BEAN, M. A., ROSEN, G. & OETTGEN, H. F. (1973) Pre-labelling Target Cells with $^{3}$H-proline as a Method for Studying Lymphocyte Cytotoxicity. *Natn. Cancer Inst., Monogr.*, **37**, 41.

CASTRO, J. E., LANCE, E. M., MEDAWAR, P. B., ZANELLI, J. & HUNT, R. (1973) Foetal Antigens and Cancer. *Nature*, **243**, 225.

COGGIN, J. H., AMBROSE, K. R. & ANDERSON, N. G. (1970) Fetal Antigen Capable of Inducing Transplantation Immunity against SV40 Hamster Tumour Cells. *J. Immunol.*, **105**, 524.

COHEN, I. R., GLOBERSON, A. & FELDMAN, M. (1971) Rejection of Tumour Allografts by Mouse Spleen Cells Sensitized *In vitro*. *J. exp. Med.*, **133**, 821.

GORCZYNSKI, R. M. (1976*a*) Autoreactivity Developing Spontaneously in Cultured Mouse Spleen Cells. I. Evidence that Cytotoxicity is Directed against Embryo-associated Antigen. *Immunology*, **31**, 607.

GORCZYNSKI, R. M. (1976*b*) Autoreactivity Developing Spontaneously in Cultured Mouse Spleen Cells. II. Comparison of Cytotoxicity of Cultured Male and Female Spleen Cells. *Immunology*, **31**, 615.

GORCZYNSKI, R. M. (1976*c*) Control of the Immune Response. Role of Macrophages in Regulation of Antibody and Cell Mediated Immune Responses. *Scand. J. Immunol.*, **5**, 1031.

GORCZYNSKI, R. M. (1976*d*) Autoreactivity Developing Spontaneously in Cultured Mouse Spleen Cells. III. Inhibition of Anti-embryo Cytotoxicity in Male T Lymphocytes by Female non-T Cells. *Immunology*, **31**, 625.

HANNA, M. G., TENNANT, R. W. & COGGIN, J. H. (1971) Suppressive Effect of Immunization with Mouse Fetal Antigens on Growth of Cells Infected with Rauscher Leukemic Virus and on Plasma-cell Tumours. *Proc. natn. Acad. Sci.*, **68**, 1748.

HÄYRY, P. & ANDERSON L. C. (1973) Sequential Responses of Mouse Spleen T Cells in Mixed Lymphocyte Culture Induced Cytolysis. *J. exp. Med.*, **141**, 508.

HELLSTRÖM, I. & HELLSTRÖM K. E. (1976) Specific Sensitization of Lymphocytes to Tumour Antigens by Co-cultivation with Peritoneal Cells Exposed to such Antigens. *Int. J. Cancer*, **17**, 748.

HOLLANDER, N., GINSBURG, H. & FELDMAN, M. (1974) *In vitro* Generation of Memory Lymphocytes Reactive to Transplantation Antigens. *J. exp. Med.*, **140**, 1057.

MACDONALD, H. R., CEROTTINI, J. C. & BRUNNER, K. T. (1974) Generation of Cytotoxic T Lymphocytes *In vitro*. III. Velocity Sedimentation Studies on the Differentiation and Fate of Effector Cells in Long-term Mixed Leukocyte Cultures. *J. exp. Med.*, **140**, 1511.

MILLER, R. G. & PHILLIPS, R. A. (1969) Separation of Cells by Velocity Sedimentation. *J. cell Physiol.*, **73**, 191.

PARMIANI, G. & LEMBO, R. (1974) Effect of Anti-embryo Immunization on Methylcholanthrene-induced Sarcoma Growth in Balb/c Mice. *Int. J. Cancer*, **14**, 555.

PEARSON, G. & FREEMAN, G. (1968) Evidence Suggesting a Relationship between Polyoma Virus-induced Transplantation Antigen and Normal Embryonic Antigen. *Cancer Res.*, **28**, 1665.

RUSSELL, S. W., GILLESPIE, G. Y., HANSEN, C. G. & COCHRANE, C. G. (1976) Inflammatory Cells in Solid Murine Neoplasm. II. Cell Types Found Throughout the Course of Moloney Sarcoma Regression or Progression. *Int. J. Cancer*, **18**, 331.

SILVERBERG, E. & GRANT, R. N. (1970) Cancer Statistics 1970. *Cancer*, **20**, 10.

TING, C. C. (1968) Failure to Induce Transplantation Resistance against Polyoma Tumour Cells with Syngeneic Embryonic Tissue. *Nature*, **217**, 858.

WEKERLE, H. (1977) *In vitro* Induction of Immunological Memory against Testicular Autoantigens. *Nature*, **267**, 357.